# Supplementary material for: The spatiotemporal distribution of Japanese Encephalitis cases in Yunnan Province, China, from 2007 to 2017
Source: PLoS One. 2020 Apr 14;15(4):e0231661. doi: 10.1371/journal.pone.0231661 (PMC7156086; doi:10.1371/journal.pone.0231661)
Supplement: Attachment — Submitted filename: rebuttal letter.docx [file pone.0231661.s004.docx]

Dear Rita Czirjak,

Thank you for your letter. It is true that the manuscript entitled "The Spatio-temporal Distribution of Japanese Encephalitis Cases in Yunnan Province, China from 2007 to 2017" was resubmitted to PLOS ONE. However, the manuscript was revised before the resubmission. The changes are as follows.

Best wishes.

Yours truly,

Xiang-hua Mao

Reviewer #1: This manuscript analyzes Japanese encephalitis virus cases in Yunnan province, China from 2007 to 2017, with the primary objective being to inform vaccination program priorities. While the study appears to be sound, the language is unclear, making it difficult to follow. It is recommended that the authors work with a writing coach or copyeditor to improve the flow and readability of the text so that a comprehensive review of the manuscript can be conducted.

Authors reply: We have changed the style of writing into America English. The errors of writing were revised as we can as possible.

Reviewer #2: The manuscript needs to be corrected by somebody fluent in English because there are numerous grammatical errors. Other general concerns include i) the absence of a space between once sentence and the next on many occasions, ii) the absence of a space between the last word of a sentence and the reference that follows on many occasions and iii) the absence of a space between subheadings in parts of the manuscript, i.e. parts of the results section. It would also be preferable if doubled-spaced text was used throughout, provided the instructions to authors allow it. All of these issues make the manuscript difficult to read.

Authors reply: We have changed the style of writing into America English, and checked the errors of writing, especially at the grammar. If there was any wordy sentence, we deleted them under the premise of keeping the original intention. We also add or remove a space or a comma if it needed, to make the text smoothly. We set doubled-spaced style for the text.

Line 23 – twice?
Authors reply: We changed the word “twice” to “two groups”, and the whole sentence was “Individuals were divided into two groups, one is less than 10 years old, and the other is greater than 10 years old”. All of the contents in the manuscript were revised.

Lines 29-30: it is not apparent after reading the manuscript whether Kunming, Zhaotong, Jinghong etc. are counties or municipalities.

Authors reply: We added a word for every place, and revised the text for these places. The whole sentence was “The top ten areas with most JE cases were Kunming city, Zhaotong city, Jinghong city, Wenshan city, Mangshi city, Puer city, Baoshan city, Dali city, Chuxiong city, and Gejiu city.”

Line 31 and elsewhere: round off to the nearest decimal place

Authors reply: We reserved one decimal place for line 31, and the contents for the same description were revised for one decimal place. The whole sentence was “The incidence declined smoothly with a peak occurring from June to September, which takes 96.1% of the total cases.”

Line 41: “there was no difference at the quality between adults and children” – what does this mean?

Authors reply: We changed the word “quality” to “quantity”, and the whole sentence was “there was no difference at the quantity between two groups.” Additionally, we corrected the same errors in the submission.

Individuals were divided into two groups: those less than 10 and those greater than 10 years old age. It is not correct to say that children were in one group and adults were in the other because. It would be preferable if there were more than two age groupings – i.e. individuals should be separated as 0-10, 11-20, 21-30, 31-40, etc. years

Authors reply: “Individuals aged 11-17 years are not adults”, that discussion of the original text is a serious flaw as pointed out. We corrected the discussion and divided the individuals into two age groups, one is 0-10, and the other is above10 years. Why do we separate the individuals in this way? Because of our goal is to clear the priorities of vaccination. We want concluded that the incidence of JE is still high at 0-10 individuals even after a large-scale vaccination.

Line 106: “China has a perfect reporting system of epidemics” – no country has a perfect reporting system
Authors reply: We changed the word “perfect” to “relevant”, and the whole sentence was “China has a relevant reporting system of epidemics and strong support of network for data collection.”

Lines 169: what are “commercial people”?
Authors reply: We changed the word “commercial people” to “merchant”, and the whole sentence was “the occupation of the other cases was medical staff, merchant, farmer or urban residents, which took 31.5% of the total.”

Line 169: what is meant by a “citizen”? Surely, every person or almost every person in this study is a citizen of China, yet it appears that children, students, farmers etc. were not considered to be citizens.
Authors reply: We changed the word “citizen” to “urban residents”, and the whole sentence was “the occupation of the other cases was medical staff, merchant, farmer or urban residents, which took 31.5% of the total.”

Line 212: it is not correct to say that “the incidence or mortality of JE decreased year by year”. For example, the number of cases in 2009 was higher than 2008
Authors reply: It is a general description for the data, however, it is not exactly. We revised that sentence as “although the incidence or mortality of JE had a downtrend in Yunnan province, China, the risk of an epidemic or an outbreak is still high.”

Figure 1: the years in x axis needed to be spaced out, as opposed to appearing a continuous text

Authors reply: Figure 1 was created again according to the patterns, the new figure is as follow.
