# Supplementary material for: The spatiotemporal distribution of Japanese Encephalitis cases in Yunnan Province, China, from 2007 to 2017
Source: PLoS One. 2020 Apr 14;15(4):e0231661. doi: 10.1371/journal.pone.0231661 (PMC7156086; doi:10.1371/journal.pone.0231661)
Supplement: Attachment — Submitted filename: Response to Reviewers.rtf [file pone.0231661.s005.rtf]

Response to the Editor

6 March 2020
Dr. Tian Wang Academic Editor
PLOS ONE

Dear Dr. Wang,

We have received the comments about this manuscript, thank you for your suggestions. We have modified the manuscript in accordance with the format requirements of PLOS ONE. The main changes are as follows:
1. Corrected the manuscript, including words and usage, to make it clear, correct, and unambiguous.
2. Adjust the manuscript format including fonts, size, line spacing, etc.
3. The uploaded pictures are reproduced in accordance with the publishing requirements, and the picture format is TIFF, 300DPI or 600 DPI.

Yours sincerely,

Xianghua Mao
Yunnan Provincial Center of Arbovirus Research
Yunnan Institute of Parasitic Diseases 


#Response to reviewer 2 
1.	Portions of the manuscript have improved compared to the first submission. Unfortunately, the manuscript still contains numerous grammatical errors throughout, which make it difficult to follow and understand the methods. Additional editing is needed.

Response: Thank you for the helpful comment. In accordance with the comment, we have asked professionals (American Journal Experts, AJE) to carefully revise the manuscript. Additionally, to make sure the statistical analysis been performed appropriately and rigorously, we also added new supporting information to ensure the conclusions.
